# Comparative analysis of the amino acid sequence of the Cry protein of *Bacillus thuringiensis *codify the toxic protein to insects of the orders Lepidoptera, Diptera and Lepidoptera/ Diptera

**DOI:** 10.1186/1753-6561-8-S4-P120

**Published:** 2014-10-01

**Authors:** Hagar Maciel, Sonia Zingaretti, Geveraldo Maciel

**Affiliations:** 1 Instituto Federal de Educação, Ciência e Tecnologia do Sul de Minas - Campus Machado, Carvalhópolis, Brazil; 2 Universidade de Ribeirão Preto - Unaerp, Ribeirão Preto, Brazil

## Background

The use of *Bt *crystal protein as a natural insecticide, applied directly to crops has been true for many years. Cry genes codifing these proteins are already are being used in transformation plants that become resistant to certain insects. The aim of this study was to compare the amino acid sequences that encode the Cry protein to insects of the orders Lepidoptera, Diptera and Lepidoptera / Diptera. We evaluated 15 sequences that were deposited in the GenBank and using bioinformatics tools to align and compare these sequences that present three domains (I, II, and III). It is assumed that changes in these domain influence the toxicity of the protein and her order. The results presented indicate the presence of specific regions and that may be related to the specificity of protein toxicity.

## Methods

The search and download all the amino acid sequences by domains: I, II and III, of the GenBank and of the program to consult "The Blast" - "Basic Local Alignment Search Tools (Altschul et al., 1997).

The sequences were divided into the following insect orders: Coleoptera, Diptera, Lepidoptera, Lepidoptera and Coleoptera, Lepidoptera and Diptera and Heminóptera.

To compare the sequences based on similarities between the genes that act on a particular species we selected 5 genes described that attack insects of the order Lepidoptera: Cry1Ba1, Cry1Ba2, Cry1Bb1, Cry1Ca1, Cry1Ca2; 5 genes of the order Diptera: Cry4Ba1, Cry4Ba2, Cry10Aa1, Cry16Aa1, Cry19Ba1 and 5 genes, whose toxin attacks insects of the order Lepidoptera and Diptera: Cry1Aa1, Cry1Aa2, Cry1Ab1, Cry1Ab2, Cry1Ac1 and by to align these sequences using the software CLUSTAW v.1.81 (Thompson et al., 1997).

## Results and conclusions

This analysis revealed the occurrence of identical sequences in the 3 domains, I = 16, II = III = 6 and 14 positions. Most of these amino acids belong to the group R nonpolar and aliphatic amino acids and not were occur amino acids positively charged R group in Domain I.

In the domains I, II and III was found that 8, 5 and 7 positions, respectively, were changes between amino acids of the same group (preserved), representing 40, 25 and 35%.

In the field I was exchanging groups of amino acids conferring differences in behavior and stability of the molecule. The exchange of amino acids at positions 192, 226 and 171 provided a more hydrophilic molecule while the exchanges of amino acids at positions 142, 49 and 87 provided a more hydrophobic molecule.

Position 224 in domain II, and domain III at position 84 was exchanged amino acids increasing the stability of proteins by promoting hydrophobic interactions in the your interior.

When has been change in the amino acid change from one group to another modifying the chemical structure of the protein, their polarity and consequently their solubility in water and its interactions with other amino acids.
